# Endothelial transcriptomic analysis identifies biomarkers of severe and cerebral malaria

**DOI:** 10.1172/jci.insight.172845

**Published:** 2023-11-22

**Authors:** Cláudia Gomes, Rosauro Varo, Miquel Duran-Frigola, Antonio Sitoe, Rubão Bila, Sonia Machevo, Alfredo Mayor, Quique Bassat, Ana Rodriguez

**Affiliations:** 1 New York University Grossman School of Medicine, New York, USA.; 2 ISGlobal, Hospital Clínic — University of Barcelona, Barcelona, Spain.; 3 Centro de Investigação em Saúde de Manhiça (CISM), Maputo, Mozambique.; 4 Ersilia Open Source Initiative, Cambridge, United Kingdom.; 5 Spanish Consortium for Research in Epidemiology and Public Health (CIBERESP), Carlos III Health Institute, Madrid, Spain.; 6 Department of Physiologic Sciences, Faculty of Medicine, Universidade Eduardo Mondlane, Maputo, Mozambique.; 7 ICREA, Pg. Lluís Companys 23, Barcelona, Spain.; 8 Pediatrics Department, Hospital Sant Joan de Déu, Universitat de Barcelona, Esplugues, Barcelona, Spain.

**Keywords:** Infectious disease, Endothelial cells, Malaria

## Abstract

Malaria can quickly progress from an uncomplicated infection into a life-threatening severe disease. However, the unspecificity of early symptoms often makes it difficult to identify patients at high risk of developing severe disease. Additionally, one of the most feared malaria complications — cerebral malaria — is challenging to diagnose, often resulting in treatment delays that can lead to adverse outcomes. To identify candidate biomarkers for the prognosis and/or diagnosis of severe and cerebral malaria, we have analyzed the transcriptomic response of human brain microvascular endothelial cells to erythrocytes infected with *Plasmodium falciparum*. Candidates were validated in plasma samples from a cohort of pediatric patients with malaria from Mozambique, resulting in the identification of several markers with capacity to distinguish uncomplicated from severe malaria, the most potent being the metallopeptidase ADAMTS18. Two other biomarkers, Angiopoietin-like-4 and Inhibin-βE were able to differentiate children with cerebral malaria within the severe malaria group, showing increased sensitivity after combination in a biomarker signature. The validation of the predicted candidate biomarkers in plasma of children with severe and cerebral malaria underscores the power of this transcriptomic approach and indicates that a specific endothelial response to *P*. *falciparum*–infected erythrocytes is linked to the pathophysiology of severe malaria.

## Graphical abstract

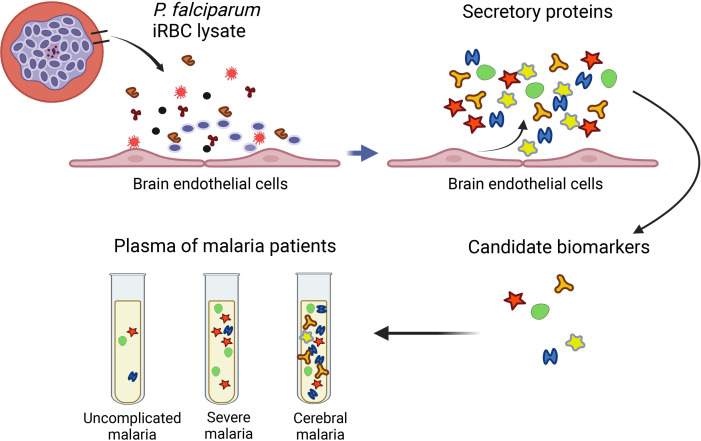

## Introduction

Despite renewed and long-standing efforts in the fight against malaria, this disease remains a major cause of global death and disability (1). In the absence of rapid treatment to clear *Plasmodium falciparum*, uncomplicated malaria (UM) may rapidly progress into severe malaria (SM), which is associated with different complications, including cerebral malaria (CM). This is one of its most feared complications, with a high risk of death (18%–30%), and is also associated with long-term cognitive and neurological deficits in up to 30%–50% of survivors (2, 3).

Around 1%–2% of patients with malaria will develop complications, but it is challenging to predict which patients are at higher risk of progressing to severe or even life-threatening disease, given the unspecificity of symptoms in early infections (4), which include fever, general malaise, headache, or vomiting (5). Malaria is most commonly diagnosed in the clinic using rapid diagnostic tests (RDTs) that were thought to reliably identify *P*. *falciparum* infections, but reports of the loss of expression of histidine-rich protein 2 (HRP2), the protein commonly used in RDTs, have evidenced high percentages of false-negative *P*. *falciparum* diagnoses in some endemic areas (6). However, in Mozambique, the area were the samples used in this work were collected, the low percentage of PfHRP2 deletions in local Plasmodium suggests that this will not greatly affect the accuracy of RDT *P*. *falciparum* diagnosis (7). Nevertheless, RDTs or conventional microscopy cannot differentiate asymptomatic, uncomplicated, and self-resolving cases from those that may progress into SM (8), which are at greatest risk of poor outcomes (9). Therefore, there is a great need to identify and validate prognostic biomarkers that can identify children at the early malaria stages with high risk of developing severe disease. Several biomarkers that are significantly associated with SM have been identified, including Angiopoietin-2, the ratio of Angiopoietin-2/Angiopoietin-1, soluble TREM-1 (sTREM-1), and specific host microRNAs, among others (10–12). However, their prognostic value has not been confirmed, since it is challenging to demonstrate their efficacy in a longitudinal study due to the low proportion of patients with malaria developing SM. Prognostic biomarkers would facilitate early and correct risk assignment of the sick patient at first encounter, allowing prioritization in cases at risk and triggering early action, consequently reducing the adverse outcomes and disability caused by SM.

In malaria-endemic areas, the specific diagnosis of CM also remains challenging. Since malaria parasitemia in the absence of any clinical symptoms is frequent among children in these areas (as high as 50%; ref. 13) and CM is, by definition, an exclusion diagnosis, it is difficult to clinically differentiate CM from other common causes of impaired consciousness or convulsions in childhood, such as bacterial meningitis, hypoglycemia, or others that can appear in the context of malaria parasitemia (14–16). The reliable diagnosis of CM will probably need the combination of several diagnostic biomarkers to achieve high broad sensitivity that can account for the individual variability among children with CM but also high specificity to exclude other causes of coma.

The difficulties to obtain a reliable pediatric CM diagnosis delay or prevent the administration of adequate treatment for CM and also confound the enrollment of patients with CM in clinical trials, since some patients with convulsions or coma induced by causes other than CM are mistakenly included, hampering the study of this syndrome. While no clear biomarker with sufficient diagnostic power to be used in the clinic exists for CM to date, some prognostic biomarkers associated with a fatal outcome in pediatric CM have been identified, such as low levels of Angiopoietin-1 (17) and high levels of Angiopoietin-2 (18), CXCL10, and CXCL4 (19).

It is becoming increasingly clear that endothelial cells and their response to the parasite and to inflammatory stimuli play a fundamental role in SM and CM pathogenesis (4, 20, 21). Previous studies have proposed candidate biomarkers for SM that are characteristic of immune cell activation, such as CXCL10, sTNFR1, or sTREM1, or of endothelial activation in response to inflammatory mediators, such as Angiopoietin-2, Angiopoietin-1, sFIt-1, or sICAM-1 (22, 23). MicroRNAs characteristic of *P*. *falciparum* activation of endothelial cells have been proposed as biomarkers of SM (11); however, protein biomarkers secreted in response to the parasite have not been studied in detail. Our previous work compared the transcriptomic response of endothelial cells to inflammatory stimuli and to *P*. *falciparum,* finding that they are largely divergent and therefore identifying a subset of endothelial genes uniquely upregulated by *P*. *falciparum* (21). Therefore, we hypothesize that the secretory profile of endothelial cells in response to the parasite would also reflect these differences, resulting in a unique set of *P*. *falciparum*–induced secreted proteins.

Here we have used this transcriptomic (RNA-Seq) analysis of human brain microvascular endothelial cells (HBMEC) (20) incubated in vitro with *P. falciparum*–infected RBCs (iRBC) (21)*,* a system that mimics the microenvironment of CM, to identify candidate biomarkers for SM and CM that are secreted in response to the parasite, rather than to inflammatory stimuli. Selection of putative secretory proteins from genes induced in HBMEC in response to *P. falciparum*–iRBCs, followed by validation in vitro and in plasma samples from a cohort of children with UM or SM (including CM), resulted in the identification of 3 potentially novel candidate biomarkers for pediatric SM: ADAM metallopeptidase with thrombospondin type 1 motif 18 (ADAMTS18), Angiopoietin-like-4 (ANGPTL4), and Inhibin-βE (INHBE). Furthermore, 2 of them, ANGPTL4 and INHBE, are also able to significantly differentiate between pediatric CM and noncerebral SM.

## Results

### Study design.

**Figure 1:**
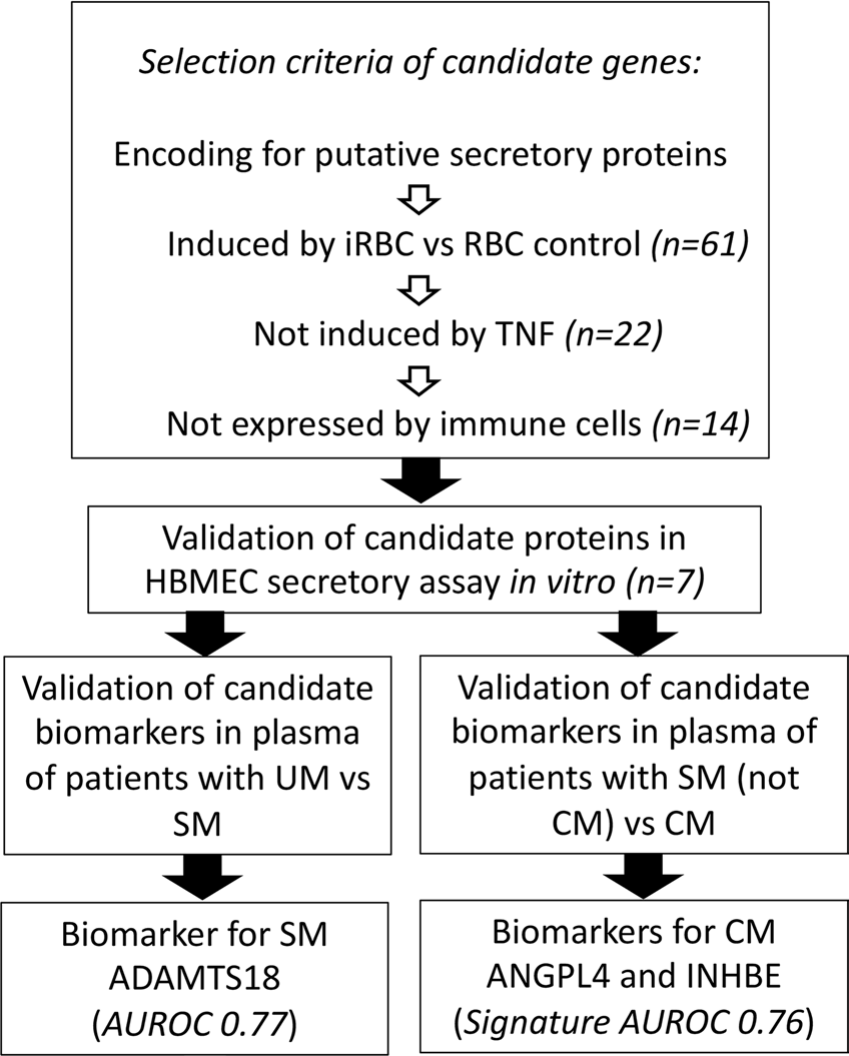
Summary of study design and results.

With the aim of identifying novel biomarkers for SM and/or CM, we have sequentially analyzed the transcriptome of HBMEC to identify genes uniquely upregulated by *P*. *falciparum*, but not by inflammatory stimuli, that encode putative secretory proteins. After confirmation that the candidate proteins are secreted in response to *P*. *falciparum* in vitro, they were validated in a pediatric cohort of malaria patients with different degrees of severity (Figure 1).

### Identification of candidate biomarkers of SM/CM from RNA-Seq results.

We have analyzed the transcriptome of HBMEC incubated with *P*. *falciparum*–infected RBC lysates (iRBCL), compared with control RBCL (21) to identify genes that are uniquely upregulated by the parasite. Since biomarker detection is performed in plasma, we first selected genes coding for proteins that are predicted to be secretory (using DAVID bioinformatic resources) (24, 25) and are also upregulated in HBMEC in response to *P*. *falciparum* iRBCL compared with control RBCL (*P* < 0.05) (21) (Supplemental Table 1; supplemental material available online with this article; https://doi.org/10.1172/jci.insight.172845DS1).

Then, we selected genes that were preferentially induced (at least 2-fold) by iRBCL compared with TNF, a potent inflammatory stimulus that is highly induced during SM and was also included in the transcriptomic analysis (21) (Supplemental Table 2). Finally, genes coding for proteins expressed in endothelial cells were selected using the Human Protein Atlas (26), eliminating the ones that are also known to be secreted by immune cells and could mask the endothelial response, leaving 14 candidate genes (Table 1).

### HBMEC secretory profile for Angiopoietin-2.

**Figure 2:**
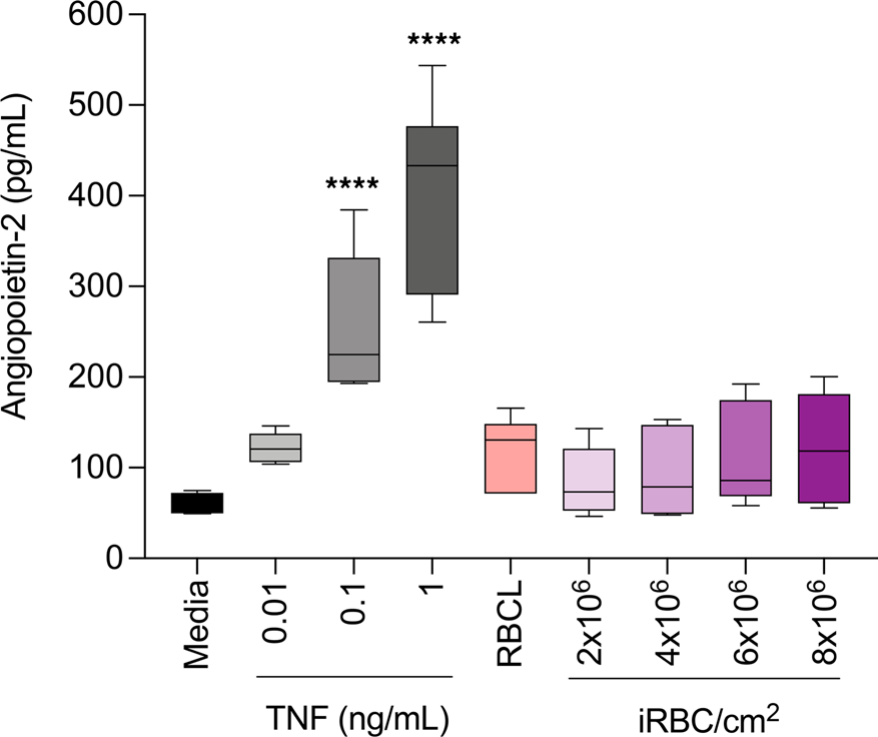
Secretion of Angiopoietin-2 is induced by TNF but not by *P*. *falciparum*–iRBCL in HBMEC. HBMEC were incubated with iRBCL at different concentrations (expressed as number of iRBC per cm^2^), RBCL (8 × 10^6^/cm^2^), or TNF at the indicated ng/mL concentrations for 24 hours before collection of the culture media and determination of the levels of angiopoietin-2 by ELISA. Results represent the mean of duplicates of 3 independent experiments with SD. Statistical significance was determined by 1-way ANOVA with Dunnett’s multiple-comparison test (****P < 0.0001).

Angiopoietin-2 is a widely accepted biomarker for SM that has already been used in multiple studies and a clinical trial (27). Angiopoietin-2 mRNA was moderately upregulated by TNF compared with control media after 6 hours in the RNA-Seq analysis (log_2_ fold change = 0.48) (21), but it did not appear in our initial selection of candidate biomarkers (Supplemental Table 1), since it was downregulated in HBMEC stimulated with iRBCL compared with control RBCL (log_2_ fold change = –0.49). We therefore investigated the secretory profile of Angiopoietin-2 in HBMEC to determine whether this marker is secreted in response to inflammatory stimuli, as previously described (28), and/or in response to *P*. *falciparum*. We observed that this biomarker is effectively secreted in response to TNF but not in response to *P*. *falciparum*–iRBCL (Figure 2), and this indicates that there are differential endothelial secretory responses depending on the nature of stimuli and confirms Angiopoietin-2 as a component of the endothelial response to inflammation. This finding also validates our approach, since we specifically selected for genes upregulated in response to *P*. *falciparum*–iRBCL.

### Validation of candidate biomarkers in vitro.

**Figure 3:**
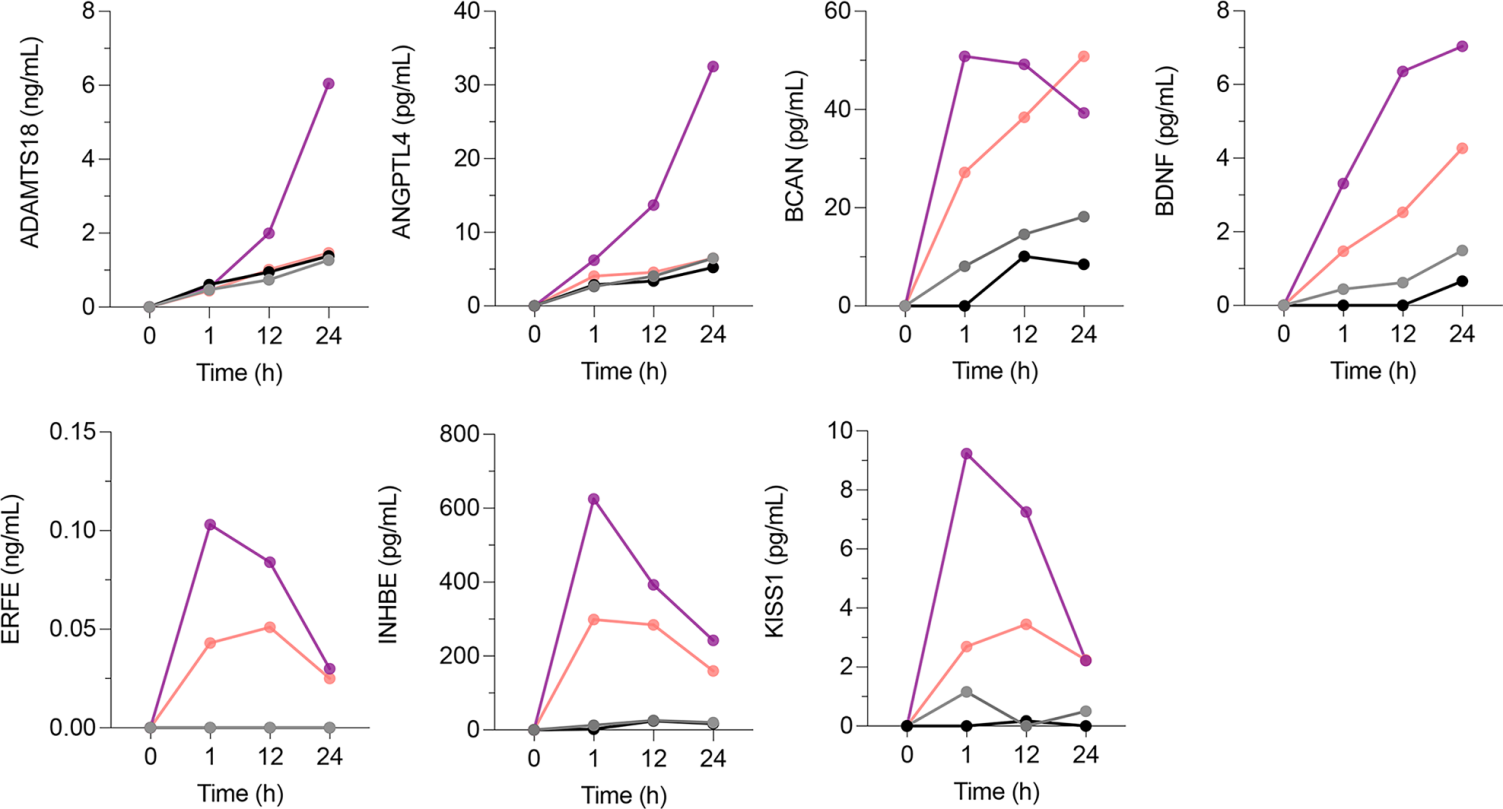
Candidate biomarker proteins secreted in response to *P*. *falciparum* but not in response to TNF. HBMEC were incubated for the indicated time points with media (black line), TNF (1 μg/mL; gray line), RBCL (8 × 10^6^ RBC/cm^2^; pink line), or *P*. *falciparum*–iRBCL (8 × 10^6^ iRBC/cm^2^; purple line). The medium of independent duplicates for each condition was collected at each time point. Levels of candidate biomarker proteins were determined by ELISA. Graphs show the average of the duplicated determinations for each time point.

We next tested whether the proteins encoded by the selected candidate biomarker genes (Table 1) are indeed secreted by HBMEC in response to *P*. *falciparum*–iRBCL. For this purpose, we determined the levels of each candidate protein in the culture media between 1 and 24 hours after incubation of HBMEC with *P*. *falciparum*–iRBCL, using uninfected RBCL as a negative control. Out of the 14 candidates, 7 proteins were confirmed to be secreted by HBMEC in response to *P*. *falciparum*–iRBCL at the protein level (Figure 3 and Table 1). Seven candidates did not meet this criterium and were not selected (Supplemental Figure 1).

### Validation of candidate biomarkers in the plasma of patients with UM and SM.

To study whether the selected candidate biomarkers could differentiate pediatric patients with SM and/or CM from patients with UM, we tested the biomarker levels in the plasma of a cohort of Mozambican children with well-characterized UM and SM. This case-control cohort includes children from 0 to 10 years (median 2.9 years) of age admitted to Manhiça District Hospital in southern Mozambique with SM (*n* = 136) or diagnosed with UM and treated as outpatients (*n* = 128). Patients with SM include children with different complications (frequently more than 1 complication in a single patient): CM (*n* = 23), prostration (*n* = 96), multiple seizures (*n* = 60), severe anemia (*n* = 35), hypoglycemia (*n* = 10), or acidosis (*n* = 53). When children with CM presented more than 1 complication, they were classified in the CM group (Table 2).

**Figure 4:**
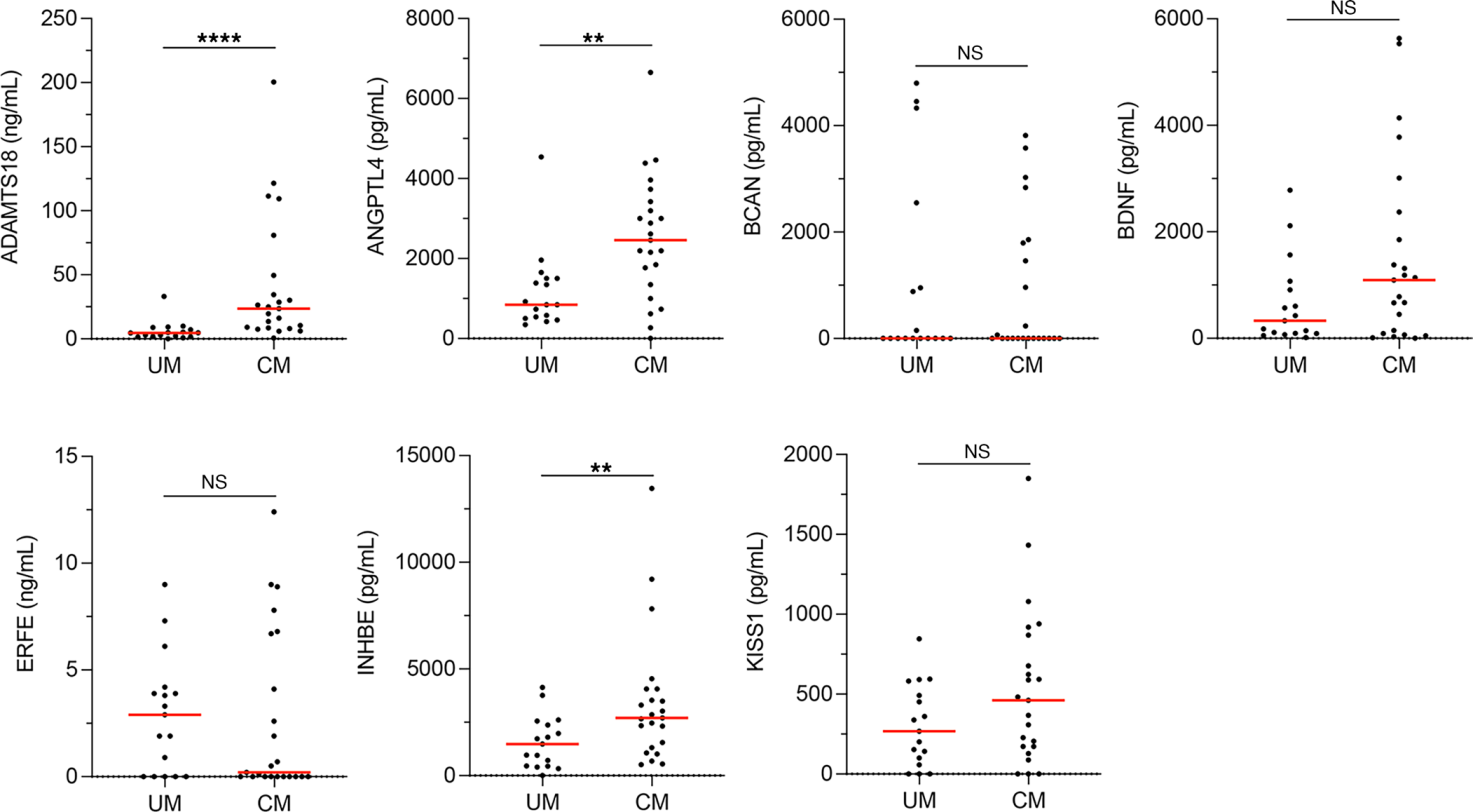
Candidate biomarkers validated in plasma samples. Comparison between the levels of the candidate biomarkers in samples from randomly selected patients with UM (*n* = 17) and all available patients with CM (*n* = 23) by ELISA. Mean level (red line). Statistical significance was determined by Mann-Whitney *U* test (**P < 0.01; ****P < 0.0001).

We first performed a preliminary selection of the 7 candidate biomarkers using specific ELISAs to determine the levels of each biomarker in a subset of the cohort samples in the least-stringent conditions — i.e., comparing children with UM versus CM. This preliminary analysis identified 3 candidate biomarkers: ADAMTS18, ANGPTL4, and INHBE (Figure 4).

**Figure 5:**
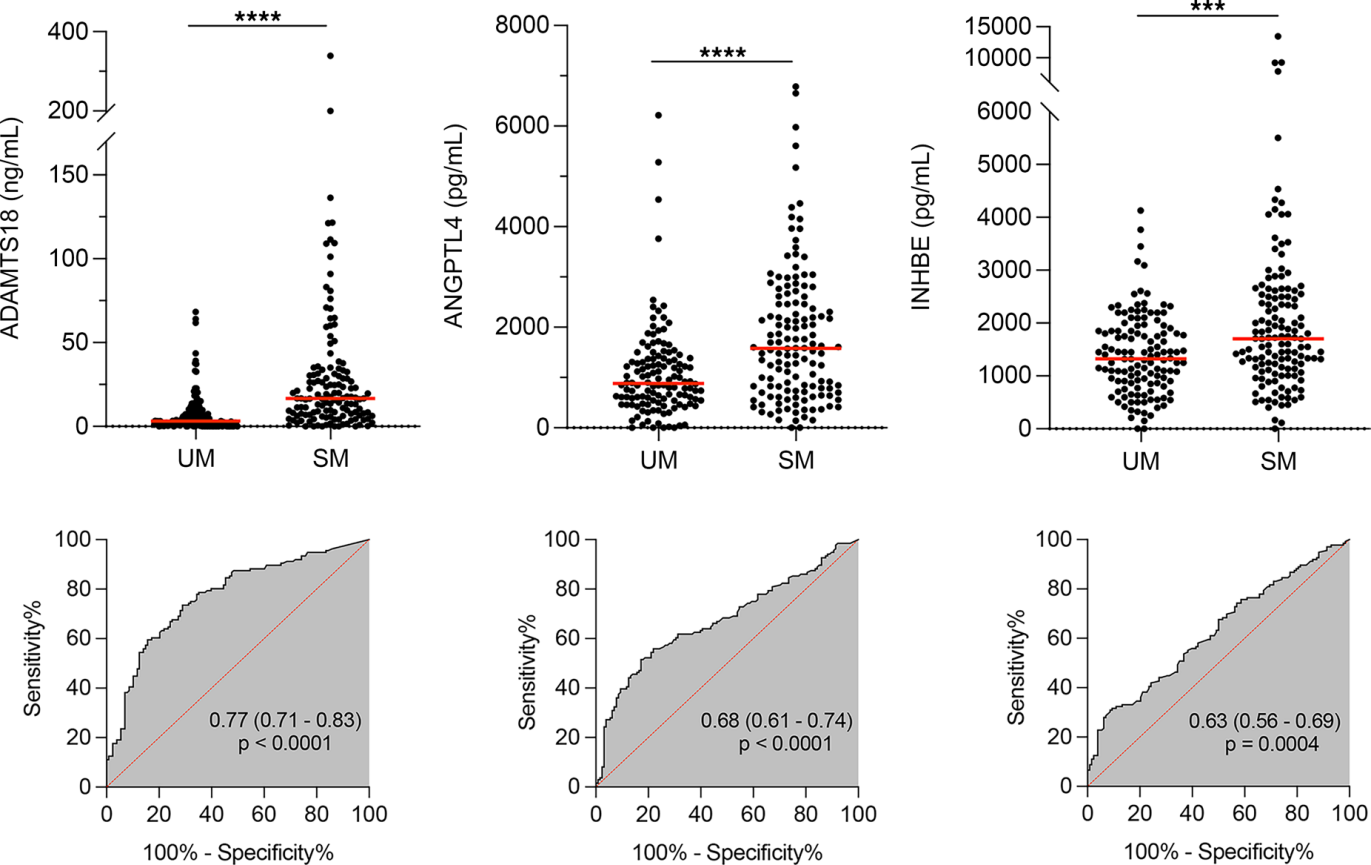
Expression levels of 3 candidate biomarkers are significantly different in UM and SM groups. Levels of the indicated biomarkers were determined in the plasma of children with UM (*n* = 128) or SM (*n* = 136) malaria by ELISA. Mean level (red line). Statistical significance was determined by Mann-Whitney *U* test (***P < 0.001; ****P < 0.0001). AUC with 95% CI and *P* value are shown.

The levels of these candidate biomarkers were then tested in the entire cohort to compare the groups with UM versus SM. We observed significant differences in the levels of ADAMTS18, ANGPTL4, and INHBE between UM and SM (Figure 5), with an AUC of 0.77 (*P* < 0.0001) for ADAMTS18; 0.68 (*P* < 0.0001) for ANGPTL4; and 0.63 (*P* = 0.0004) for INHBE. Moreover, we observed a significant correlation between ADAMTS18 and ANGPTL4 levels across the cohort (Spearman’s ρ = 0.41, *P* < 10^–11^). To a lesser extent, INHBE levels were also correlated with these biomarkers (ADAMTS18 ρ = 0.31, *P* < 1 × 10^–6^; ANGPTL4 ρ = 0.18, *P* < 4 × 10^–3^). As expected because of the correlation between them, the combination of these candidates into a single biomarker signature did not improve AUC.

### Relation of candidate biomarkers to P. falciparum biomass and malaria complications.

We also determined the relation of the candidate biomarkers to *P*. *falciparum* biomass, measured as levels of HRP-2, since malaria severity is strongly associated with this parameter (29). We observed that ANGPL4, the highest upregulated gene by *P*. *falciparum*–iRBCL (Table 1), correlated more strongly with HRP-2 (Spearman’s ρ = 0.43, *P* < 0.0001), while ADAMTS18 had a weaker correlation (Spearman’s ρ = 0.28, *P* < 0.0001), while INHBE was not significantly correlated. No correlation was found between any of the candidate biomarkers and circulating parasitemia determined by quantitative PCR (qPCR).

We also analyzed the relation of the candidate biomarkers with other malaria complications, finding a significant association of the 3 candidate biomarkers with neurological complications such as CM and prostration. ADAMTS18 and ANGPTL4 were also correlated with acidosis and severe anemia. Jaundice was only associated with ANGPTL4, and multiple seizures was associated with ADAMTS18 and INHBE (Table 3).

### A biomarker signature for pediatric CM.

**Figure 6:**
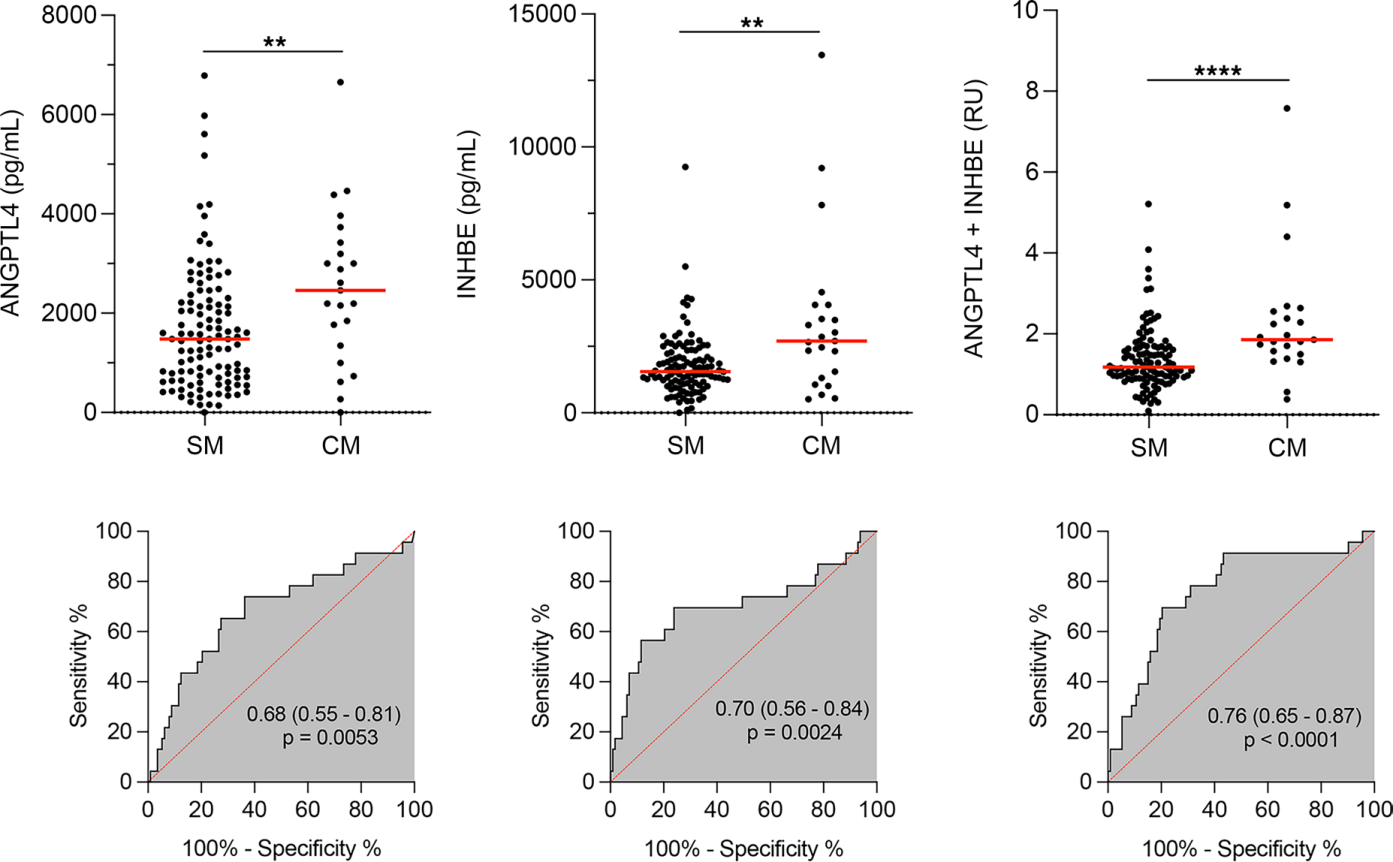
Expression levels of 2 candidate biomarkers are significantly different in SM (noncerebral) and CM groups. Levels of the indicated biomarkers were determined in the plasma of children with severe noncerebral (SM; *n* =113) or cerebral (CM; *n* = 23) malaria by ELISA. Mean level (red line). Relative units (RU) were calculated for each determination dividing by the average value of the control (SM) group. The highest of ANGPTL4 and INHBE values expressed in RU were selected and compared between SM and CM groups. Statistical significance was determined by Mann-Whitney *U* test (**P < 0.01; ****P < 0.0001). AUC with 95% CI and *P* value are shown.

We next determined whether the candidate biomarkers could differentiate between children with CM within the group with SM. Further analysis of the 2 candidate biomarkers that were significantly associated with CM, ANGPTL4 and INHBE, showed an AUC of 0.68 (*P* < 0.005) and 0.70 (*P* < 0.002), respectively (Figure 6). ADAMTS18 did not associate significantly with CM (AUC of 0.63, *P* = 0.0574). Analysis of the correlation between the levels of ANGPTL4 and INHBE showed that these 2 candidate biomarkers were not significantly correlated among SM cases (ρ = 0.17, *P* = 0.18); combining both biomarkers in a logistic regression model (CM, ANGPTL4 **+** INHBE) resulted in a good model fit (significant log-likelihood ratio [LLR], *P* < 6 × 10^–4^); and using a LLR test showed that the ANGPTL4 + INHBE model was significantly better than the individual models (ANGPTL4 + INHBE versus INHBE, *P* < 0.05; ANGPTL4 + INHBE versus ANGPTL4, *P* < 0.004). Collectively, these observations indicate that INHBE and ANGPTL4 are uncorrelated biomarkers and could serve as independent predictors, increasing the power when combined in a biomarker signature. A combined analysis of these 2 biomarkers selecting the highest relative value of each one (Figure 6) resulted in an increased discriminative power (AUC = 0.76; *P* < 0.0001).

## Discussion

Here, we characterized the secretory response to *P*. *falciparum* of human brain endothelial cells with the aim to identify novel biomarkers for pediatric SM and CM. Previously characterized candidate biomarkers for SM are predominantly molecules secreted by immune or endothelial cells in response to inflammatory stimuli (23). However, in *P*. *falciparum* malaria, there is a strong correlation between parasite biomass and disease severity (29), suggesting that *P*. *falciparum*–iRBC and/or circulating parasite–derived factors contribute to the different malaria complications. This appears more evident during CM, where densely packed capillaries with adhered *P*. *falciparum*–iRBC jeopardize blood flow and allow for the generation of microenvironments where parasite-derived factors can accumulate in high concentrations upon the rupture and release of the iRBC contents at the end of the erythrocytic infection cycle (20). During CM, cytoadhesion of *P*. *falciparum* iRBC is considered a requirement for the accumulation of iRBC in brain capillaries, leading to the disruption of the brain endothelial barrier. However, in vitro models of disruption of brain endothelial cell barrier function do not require cytoadherent iRBC (here we used noncytoadherent 3D7 *P*. *falciparum* strain), since the rupture and release of densely packed iRBC contents in close contact with endothelial cells is sufficient to cause loss of barrier integrity (20). The validation of the candidate biomarkers selected using the in vitro model in the plasma of children with SM and CM suggests that the microenvironent artificially generated in vitro (HBMEC cell line incubated with lysates of noncytoadherent iRBC) is, to some extent, similar to the conditions of SM and CM in patients. A broader secretory response may be observed using cytoadherent whole iRBC incubated with primary brain endothelial cells.

Since the transcriptomic endothelial response to inflammatory mediators in vitro is substantially different to the response to *P*. *falciparum*–iRBC (21), we designed our approach to specifically identify molecules secreted in response to *P*. *falciparum*, which resulted in the identification of candidate biomarkers for SM and CM. We identified specific proteins that are secreted by endothelial cells in response to *P*. *falciparum*–iRBC, but not in response to TNF, indicating that there is a different endothelial secretory profile depending on whether the stimulus is the parasite versus inflammatory mediators. The validation of these candidates in the plasma of children with SM, and more importantly with CM, suggests that there is a specific host endothelial response to *P*. *falciparum*–iRBC during infection, different from the inflammatory response.

Before further validation in the clinic, the candidate biomarkers would need to be tested for their specificity in the identification of patients with SM and/or CM compared with other infections or causes of coma that frequently confuse these diagnoses (14, 15). In particular, ADAMTS18, a metallopeptidase, may be also present in sepsis patients, where this family of proteins is upregulated (30). However, it is likely that the biomarkers induced specifically by *P*. *falciparum* iRBC that were selected in this work may present higher specificity for CM compared with inflammatory biomarkers, since CM and coma induced by other infectious diseases present similar inflammatory profiles (31).

The biological function of the 3 candidate biomarkers, ADAMTS18, ANGPTL4, and INHBE, has not been extensively characterized, and they have not been described before in the context of malaria. However, it is known that ADAMTS18 and ANGLP4 are required for angiogenesis and appear to play a role in the regulation of endothelial permeability (32, 33), which opens the possibility that they might play a role in CM pathology. ADAMTS18 is also highly expressed in the placenta, suggesting that it may also constitute a candidate biomarker for placental malaria. Little is known about the function of INHBE in the context of endothelial cells, but the significant association of this protein with CM and neurological complications— together with lack of association with SM in general, other nonneurological complications (acidosis, anemia, or jaundice), and *P*. *falciparum* biomass — suggests that it may be specific to brain endothelial damage, to some extent. Indeed, the lack of correlation with the other CM candidate biomarker, ANGPL4, is an important characteristic that contributed to increasing the potency of the proposed CM biomarker signature.

Interestingly, neuregulin-1 (NRG1) transcription was upregulated in HBMEC by *P. falciparum*–iRBCL, and this would be in line with previous results showing that this protein is elevated during CM in mice, implicating it in endothelial protection by regulating intracellular signaling (34, 35).

The previously characterized biomarkers for SM reach similar levels of sensitivity and specificity as the ones reported here, with Angiopoietin-2 (AUC = 0.71–0.83; ref. 10) being the leading candidate. It is likely that the development of an effective diagnostic tool for SM and CM would require the combination of several biomarker candidates in a signature that could improve prognosis and diagnosis of SM in the clinic. The candidates presented here should be validated in different geographical and age-range cohorts, especially since important differences in the pathogenesis of CM in children and adults have been observed (36). The association of the candidate biomarkers described here with already-identified biomarkers should be analyzed to determine the possibility of combination in biomarker signatures with increased predictive value. Additionally, the selected biomarkers should be studied in longitudinal cohorts to determine their value as prognostic biomarkers. However, in malaria endemic areas, most childhood episodes of fever are due to uncomplicated or self-limited infections, and only a small proportion (<1%) are potentially life threatening (37); this increases enormously the enrollment size of any study aiming to identify prognostic biomarkers.

Considering that endothelial cells during malaria are frequently exposed at the same time to the 2 types of stimuli analyzed here, *P*. *falciparum* and inflammatory mediators, expanding the search for potential biomarkers to include molecules secreted in response to both stimuli simultaneously may identify novel biomarker candidates. Similarly, the combination of secreted proteins and other biomolecules such as microRNAs may also contribute to an increase in the predictive power of biomarker signatures.

In conclusion, transcriptomic analysis of brain endothelial cells in vitro allowed for the identification of biomarkers for SM and CM that were validated in patients with malaria, showing the similarities of the endothelial in vitro model and the patient’s vascular microenvironment and opening an alternative pathway for biomarker identification.

## Methods

### Cell culture.

HBMECs were immortalized as previously described (38) and cultured in endothelial cell basal medium (ECM) (ScienCell) supplemented with 5% FBS (ScienCell), 1% endothelial cell growth factor supplement (ECGS) (ScienCell), and 1% penicillin-streptomycin solution (PS) (ScienCell) at 37°C in 5% CO_2_. HBMECs were seeded for experiments until they reached 90%–95% confluence in ECM supplemented with 0.5% FBS, 0.1% ECGS, and 1% PS.

### P. falciparum culture, isolation, and lysate preparation.

*P*. *falciparum* 3D7 was cultured in erythrocytes (Interstate Blood Bank) at 5% hematocrit in RPMI 1640 (Corning) supplemented with 25 mM HEPES (Thermo Fisher Scientific), 25 mM sodium bicarbonate (MilliporeSigma), 0.5 mM hypoxanthine (MilliporeSigma), 0.5% Albumax II (Thermo Fisher Scientific), and 10 mg/mL gentamicin (Thermo Fisher Scientific) at 37°C in a gas mixture of 5% O_2_, 5% CO_2_, and 90% N_2_. Parasite cultures were synchronized using 5% sorbitol (MilliporeSigma). *P*. *falciparum*–iRBCs at the schizont stage were isolated from highly synchronous cultures using magnetic columns (LD MACS separation columns; Miltenyi Biotec). Uninfected RBC lysates (RBCLs) or iRBCLs were generated by 10 freeze-thaw cycles using liquid nitrogen and a 37°C water bath.

The density of iRBCs incubated over the HBMEC monolayers ranged from 2 × 10^6^ to 8 × 10^6^ iRBCs/cm^2^, where 8 × 10^6^ iRBCs/cm^2^ was estimated to be equivalent to 2 layers of iRBCs over the HBMEC monolayer (21).

### HBMEC protein secretion profiles.

In total, 10,500 HBMECs were seeded in 96-well plates and incubated for 24 hours before adding the stimuli. Culture media were collected after 1, 12, and 24 hours from duplicated independent wells, centrifuged at 200*g* for 5 minutes at room temperature, and used for ELISA determination of the different candidate biomarkers. The criteria to select a candidate were that the levels of secreted protein in response to iRBCL were at least 80% higher than those in response to RBCL and to TNF at 1 time point. Responses lower than 5 pg/mL were not considered.

### ELISA determination of candidate biomarkers.

Either samples from HBMEC culture media in different conditions or plasma from children in the cohort were used to determine the levels of the different candidate biomarkers using commercial sandwich ELISA kits (Angiopoietin-2 and REN, R&D Systems; ADAMTS18, Novus Biologicals; ANGPTL4, Abcam; BCAN, Boster Bio; BDNF, Abcam; NRG1, Abbexa; and ADM2, ERFE, INHBE, KISS1, LCN6, RELN, STC2 and TUFT1, MyBiosource).

### Study population.

The plasma samples were collected in 2 case-control studies conducted in Manhiça District in southern Mozambique during 2006 (*n* = 120) and 2014 (*n* = 148). Samples were collected from children < 10 years of age diagnosed with clinical malaria (axillary temperature ≥ 37.5°C and asexual *P*. *falciparum* parasitemia ≥ 500/μL), either outpatient diagnosed with UM (no further complications) or admitted to Manhiça District Hospital for SM. Cases of SM were defined as patients with a clinical diagnosis of malaria and an asexual *P*. *falciparum* parasitemia > 0 parasites/μL by microscopic examination of Giemsa-stained blood smears, and fulfilling at least 1 of the following criteria: CM (Blantyre Coma Score ≤ 2), severe anemia (packed cell volume < 15% or hemoglobin < 5 g/dL), acute respiratory distress (chest indrawing and/or deep breathing), acidosis (hyperlactatemia; lactate > 5 mM), jaundice (bilirubin > 60 μmol/L), prostration (inability to sit or breastfeed in children old enough to do so), hypoglycemia (blood glucose < 2.2 mM), and multiple seizures (≥ 2 convulsions in the preceding 24 hours). Children with positive bacteremia were excluded. Children with SM were treated according to Mozambican national guidelines with parenteral quinine in 2006 or parenteral artesunate (complemented with an oral artemisinin–based combination therapy) in 2014, and those with UM were treated with a combination of oral amodiaquine and sulfadoxine-pyrimethamine (Fansidar, Roche Pharma) in 2006 or with artemether-lumefantrine (Coartem, Novartis) in 2014. Quantification of parasitemia was also performed by qPCR targeting the *P*. *falciparum* 18S rRNA gene (11).

### Sample collection and processing.

Heparinized blood (10 mL) from study participants was collected and processed within 2 hours. After centrifugation at 200*g* for 10 minutes at 4°C, plasma was stored at −20°C. The 2014 study was conducted identically to the 2006 study; with the only differences being that the anticoagulant was EDTA in the later cohort. Analysis of the medians of each biomarker in each patient group (NS, SM, and CM) was compared between both studies, showing differences < 7% of the maximum value between both studies.

### Biomarker signature combination for SM and CM.

Power transformation on the values was performed. Based on this, the maximum value for each biomarker was selected for each patient and was used to calculate the signature. Analysis using Support Vector Classifiers, Logistic regression, and TabPFN classifier (39) methods was also performed but did not significantly increase the signature value compared with the simple maximum value selection.

### Statistics.

Statistical analyses were performed on Prism 9 (GraphPad Software). The Shapiro-Wilk test was used to test whether the data followed normal distribution. The specific statistical tests used are detailed in the figure legends. The exact statistical tests used are detailed in the figure legends, and *P* < 0.05 was considered significant. Differences in continuous variables were analyzed using the Mann-Whitney *U* test with Bonferonni correction to adjust for multiple testing. Differences in categorical variables were assessed using Fisher’s exact test or Pearson’s χ^2^ test, as appropriate.

### Study approval.

Written informed consent was obtained from parents or guardians of study participants prior to participation. Ethical approval was granted by The National Mozambican Ethical Review Committee (Mozambique) and Hospital Clínic (Barcelona, Spain).

### Data availability.

The RNA-Seq data used in this manuscript were described before (21) and are available in the Gene Expression Omnibus (GEO) database (accession no. GSE211439). Values for all data points in graphs are reported in the Supporting Data Values file.

## Author contributions

CG, AR, MDF, and RV designed research studies; CG conducted experiments; AS, RB, and SM collected patient samples; RV, QB, and AM designed patient studies; CG, MDF, and AR analyzed data; and AR, CG, RV, MDF, and QB wrote the manuscript.

## Supplementary Material

Supplemental data

Supporting data values

## Figures and Tables

**Table 3:**
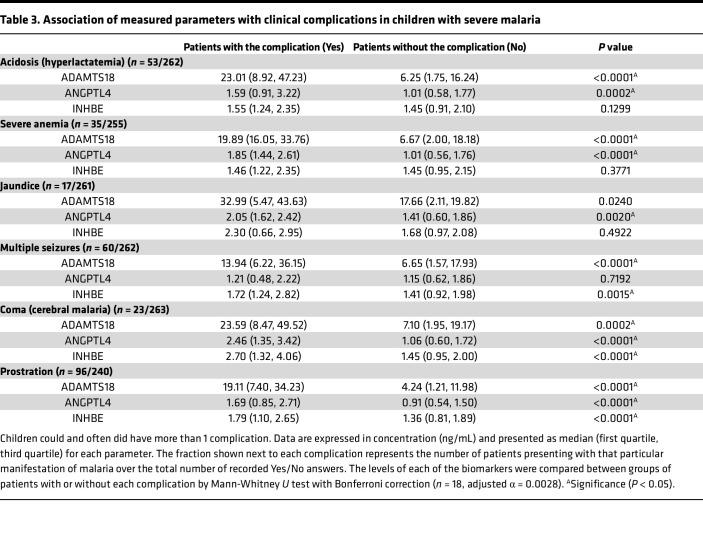
Association of measured parameters with clinical complications in children with severe malaria

**Table 2:**
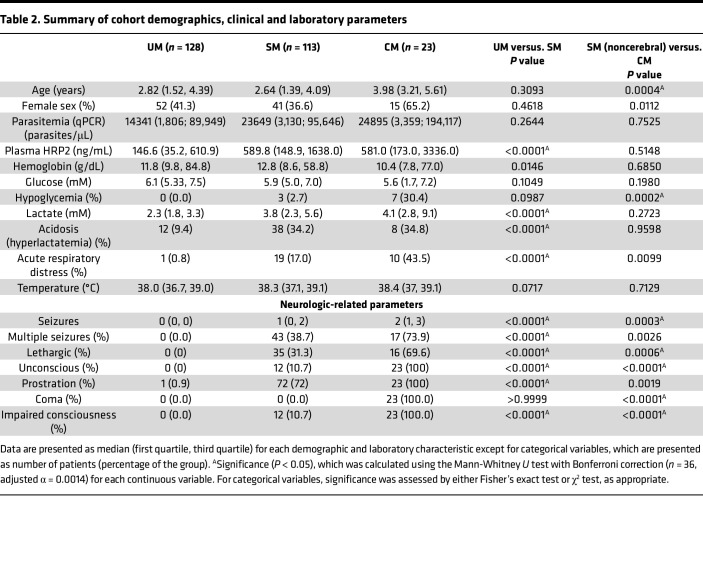
Summary of cohort demographics, clinical and laboratory parameters

**Table 1:**
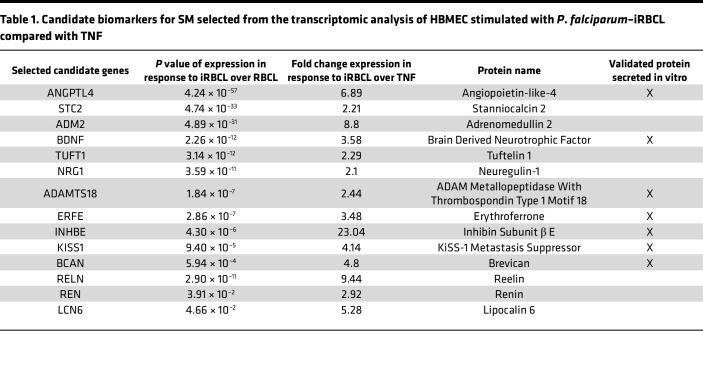
Candidate biomarkers for SM selected from the transcriptomic analysis of HBMEC stimulated with *P*. *falciparum*–iRBCL compared with TNF

## Version 1 10/03/2023

In-Press Preview

## Version 2 11/22/2023

Electronic publication
