# Supplementary material for: Endothelial transcriptomic analysis identifies biomarkers of severe and cerebral malaria
Source: JCI Insight. 2023 Nov 22;8(22):e172845. doi: 10.1172/jci.insight.172845 (PMC10721316; doi:10.1172/jci.insight.172845)
Supplement: Supplemental data [file jciinsight-8-172845-s104.pdf]

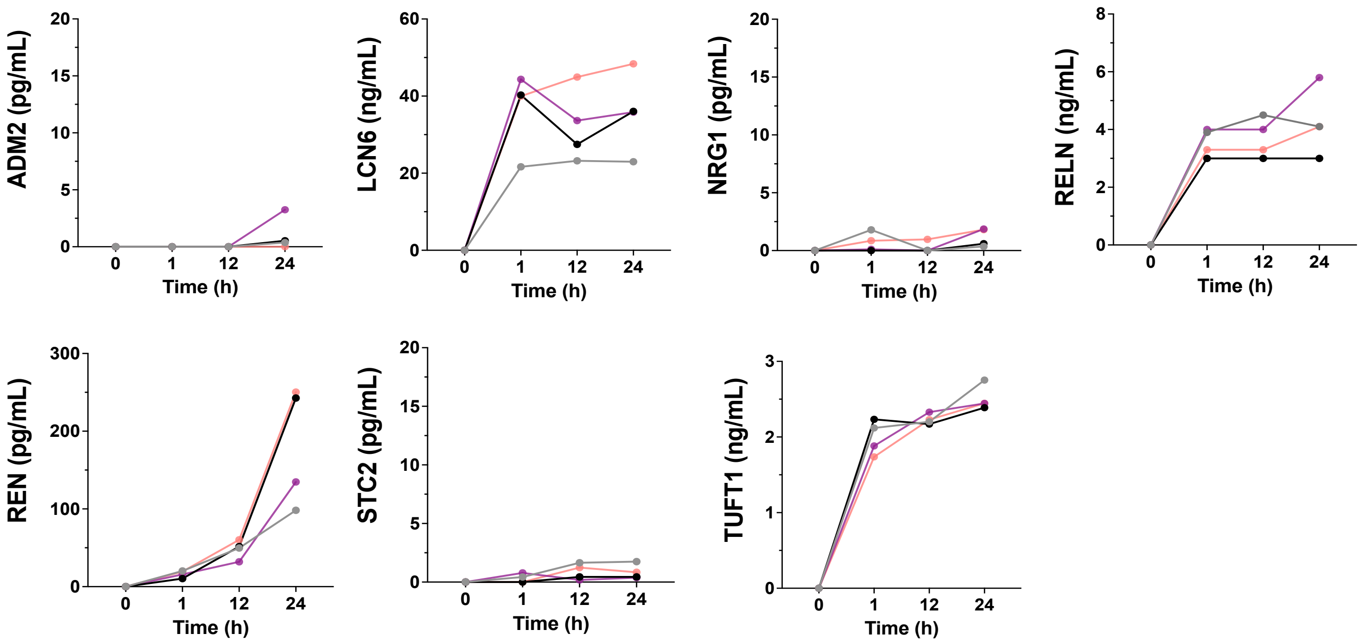

Figure S1. Candidate biomarker proteins that are not differentially secreted in response to *P. falciparum* and TNF-α. HBMEC were incubated for the indicated time points with media (black line), TNF-α (1 µg/mL; gray line), RBCL (8 x 10^6 RBC/cm^2; pink line) or *P. falciparum*-iRBCL (8 x 10^6 iRBC/cm^2; purple line). Levels of candidate biomarker proteins were determined in the medium of duplicated culture wells by ELISA.

Gene symbol	Protein	*P* adjusted
ADAMTS4	ADAM metallopeptidase with thrombospondin type 1 motif 4	1.81E-09
ADAMTS18	ADAM metallopeptidase with thrombospondin type 1 motif 18	1.84E-07
ADM2	Adrenomedullin 2	4.89E-31
ANGPTL4	Angiopoietin like 4	4.24E-57
BMP2	Bone morphogenetic protein 2	1.69E-08
BDNF	Brain derived neurotrophic factor	2.26E-12
BCAN	Brevican	0.000594018
CCL2	C-C motif chemokine ligand 2	2.24E-27
CCL20	C-C motif chemokine ligand 20	4.79E-09
CXCL1	C-X-C motif chemokine ligand 1	8.50E-19
CXCL2	C-X-C motif chemokine ligand 2	6.66E-16
CXCL3	C-X-C motif chemokine ligand 3	4.48E-10
CXCL6	C-X-C motif chemokine ligand 6	2.95E-09
CXCL8	C-X-C motif chemokine ligand 8	3.15E-28
CX3CL1	C-X3-C motif chemokine ligand 1	0.013416408
C12orf73	Chromosome 12 open reading frame 73	4.52E-07
F3	Coagulation factor III, tissue factor	1.31E-09
CSF2	Colony stimulating factor 2	0.023109419
CSF3	Colony stimulating factor 3	0.038339442
C3	Complement C3	0.022496086
CRELD2	Cysteine rich with EGF like domains 2	2.61E-16
EBI3	Epstein-Barr virus induced 3	0.000496793
ESM1	Endothelial cell specific molecule 1	9.03E-05
ERFE	Erythroferrone	2.86E-07
FGF5	Fibroblast growth factor 5	5.26E-09
FST	Follistatin	7.76E-09
GDF15	Growth differentiation factor 15	2.23E-41
HBEGF	Heparin binding EGF like growth factor	4.33E-09
INHBA	Inhibin beta A subunit	0.014380204
INHBE	Inhibin beta E subunit	4.30E-06
IL1A	Interleukin 1 alpha	4.15E-14
IL1B	Interleukin 1 beta	3.38E-06
IL11	Interleukin 11	0.023191129
IL23A	Interleukin 23 subunit alpha	0.009203239
IL32	Interleukin 32	0.003289992
IL6	Interleukin 6	1.92E-24
KISS1	KiSS-1 metastasis-suppressor	9.40E-05
KITLG	KIT ligand	4.01E-10
LAMB3	Laminin subunit beta 3	0.000297035
LAMC2	Laminin subunit gamma 2	0.020668401
LIF	Leukemia inhibitory factor	1.64E-07
LCN6	Lipocalin 6	0.04657047
MMP10	Matrix metallopeptidase 10	1.06E-08
MANF	Mesencephalic astrocyte derived neurotrophic factor	2.99E-28
NRG1	Neuregulin 1	3.59E-11
PTHLH	Parathyroid hormone like hormone	0.036146238
PTX3	Pentraxin 3	4.58E-11
RELN	Reelin	2.90E-11
REN	Renin	0.039139873
RSPO3	R-spondin 3	0.009993491
SEMA3A	Semaphorin 3A	5.38E-16
SERPINB2	Serpin family B member 2	0.000160109
STC2	Stanniocalcin 2	4.74E-33
SDC4	Syndecan 4	7.65E-09
TGFB2	Transforming growth factor beta 2	3.57E-05

TUFT1	Tuftelin 1	3.14E-12
TNFSF15	Tumor necrosis factor superfamily member 15	4.60E-10
TNF	Tumor necrosis factor	0.000650306
VEGFA	Vascular endothelial growth factor A	4.02E-23
VEGFC	Vascular endothelial growth factor C	5.19E-16
VSTM1	V-set and transmembrane domain containing 1	0.001304636

Table S1 – HBMECs significantly upregulated genes encoding for predicted secreted proteins in response to iRBCL compared to RBCL (*P* adjusted <0.05).

Gene symbol	Protein	Foldchange iRBCL vs TNF-α
ADAMTS18	ADAM metallopeptidase with thrombospondin type 1 motif 18	2.44
ADM2	Adrenomedullin 2	8.80
ANGPTL4	Angiopoietin like 4	6.89
BDNF	Brain derived neurotrophic factor	3.58
BCAN	Brevican	4.80
C12orf73	Chromosome 12 open reading frame 73	2.74
ERFE	Erythroferrone	3.48
GDF15	Growth differentiation factor 15	4.19
INHBE	Inhibin beta E subunit	23.04
IL1A	Interleukin 1 alpha	3.75
IL11	Interleukin 11	5.58
IL6	Interleukin 6	3.83
KISS1	KiSS-1 metastasis-suppressor	4.14
KITLG	KIT ligand	2.79
LIF	Leukemia inhibitory factor	2.05
LCN6	Lipocalin 6	5.28
NRG1	Neuregulin 1	2.10
RELN	Reelin	9.44
REN	Renin	2.92
STC2	Stanniocalcin 2	2.21
TUFT1	Tuftelin 1	2.29
VEGFA	Vascular endothelial growth factor A	2.44

Table S2 – HBMECs genes from Table S1 induced at least 2-fold in response to incubation with iRBCL compared to TNF-α.
